# Enhancing Ultraviolet Stability and Operational Durability of Perovskite Photodetectors by Incorporating Chlorine into Thermally-Switchable Tautomeric Passivators

**DOI:** 10.1007/s40820-025-02015-5

**Published:** 2026-01-05

**Authors:** Yong Wang, Guangsheng Liu, Feng Lin, Yuqin Hu, Niu Lai, Junhong Lv, Shuming Ye, Jie Yang, Rongfei Wang, Feng Qiu, Yu Yang, Wenhua Zhang, Chong Wang

**Affiliations:** 1 Yunnan Key Laboratory for Micro/Nano Materials & Technology, School of Materials and Energy, Yunnan University, Kunming, 650500 People’s Republic of China; 2 School of Metallurgy and Materials, Wenshan University, Wenshan, 663099 People’s Republic of China; 3 Yunnan Key Laboratory of Electromagnetic Materials and Devices, School of Materials and Energy, Yunnan University, Kunming, 650091 People’s Republic of China

**Keywords:** Perovskite photodetector, Tautomer passivator, Surface defect passivation, Ultraviolet absorbent

## Abstract

UV-absorbing additives have recently been demonstrated to be effective interfacial modifiers that simultaneously enhance the UV stability and crystallization of halide perovskite. However, the underlying mechanisms concerning UV absorption, defect passivation, and efficacy optimization of these additives remain unresolved. Herein, two UV tautomeric absorbers (UV320 and UV327) are selected as defect-passivators for perovskites. The keto–enol tautomeric evolution processes and corresponding defect passivation performance/mechanism of both the original molecules and their tautomers are thoroughly compared and elucidated through experimental characterizations and density functional theory calculations. The additional carbonyl (–C=O) groups generated through the keto–enol tautomeric process triggered by the Cl atom in UV327 ultimately provide superior chemical coordination and enhanced defect-passivation capability compared to the original counterparts. Moreover, the versatility of K-UV327 is further demonstrated by its optimization of SnO_2_ film quality, interfacial energy band alignment, charge extraction efficiency, and defect state suppression. The photodetector optimized by UV327’s tautomer achieves an ultralow dark current density of 3.22 × 10^−10^ A cm^−2^, an enhanced linear dynamic range of 94.14 dB, and a fast response time of 23.35/26.19 μs. Notably, unencapsulated devices maintain a stable response at 3900 Hz following 300 h exposure to 40% ± 5% relative humidity and 30 h UV irradiation.

**Supplementary Information:**

The online version contains supplementary material available at 10.1007/s40820-025-02015-5.

## Highlights

- The tautomeric UV absorbers (UV320/UV327) in perovskites reveal keto–enol tautomerism, generating extra –C=O groups to enhance defect passivation.
- The Cl atom in UV327 drives tautomerism, providing superior –C=O coordination, which optimizes SnO_2_ energy bands/charge extraction, resulting in a dark current of 3.22 × 10^−10^ A cm^−2^ and a response time of 23.35/26.19 μs.
- Unencapsulated devices maintained 3900 Hz response after 300 h humidity (40 ± 5% RH) and 30 h UV stress, with 94.14 dB linear dynamic range.

**Supplementary Information:**

The online version contains supplementary material available at 10.1007/s40820-025-02015-5.

## Graphical abstract

**Figure:**
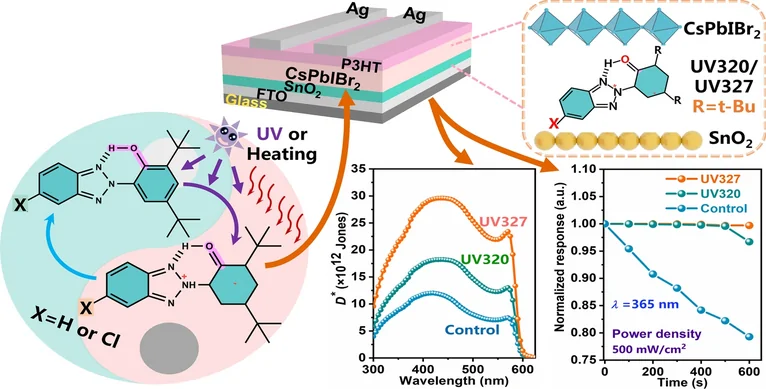

## Introduction

Due to their outstanding physical properties, all-inorganic lead halide perovskites with the chemical formula CsPbX_3_ have attracted significant attention for optoelectronic applications, such as solar cells [1–7], light-emitting diodes (LEDs) [8–10], photodetectors (PDs) [11–20], lasers, and imaging [21, 22]. Among these, PDs, which convert light into electrical signals, are essential for optical communication, biomedicine, space exploration, and imaging [23]. Nevertheless, due to the chemical instability of perovskites under moisture, light, and heat exposure, solution-processed perovskite-based PDs have not achieved the widespread adoption initially predicted by the industry [24, 25]. One primary source of instability stems from the presence of numerous defects in perovskite lattices, including uncoordinated Pb^2+^, halogen vacancies (V_X_), and Pb-I antisites (PbI_3_^−^), which act as non-radiative recombination centers and ionic migration pathways, ultimately degrading device performance [2–4, 14, 26].

Leveraging Lewis acid–base interactions and hydrogen bonding, incorporating Lewis base-functionalized electron-donating organic molecules (e.g., –CN, –NH_2_, –Cl, –C=O) into perovskite films remains an effective and widely used approach for trap-state passivation [1–4, 24, 27–30]. These functional groups can donate lone pair electrons to undercoordinated positive charge centers, such as Lewis acidic Pb^2+^, forming coordinate bonds and reducing trap states. Notably, bifunctional Lewis base/proton-donor molecules (e.g., –OH, –NH_2_) exhibit strong passivation via coordinating undercoordinated Pb^2+^, forming H-bonds with I^−^, and passivating the other cationic and anionic defects. Furthermore, when engineered into a conjugated molecular framework, particularly aromatic systems, this dual functionality significantly amplifies passivation effectiveness through synergistic π-π conjugation between carbon–carbon double bonds (–C=C) and p-π conjugation linking –C=C to carbonyl (–C=O) or imine (–C=N) groups. Additionally, incorporating extended conjugated system molecular structures can also further enhance the stability of the passivator. However, the encapsulated devices remain susceptible to prolonged UV exposure, which leads to perovskite decomposition and performance degradation [31–36]. Consequently, significant efforts focus on the strategic selection of UV-absorbing additives containing Lewis base functional groups. For instance, Zhao et al. [35] demonstrated that 2-hydroxy-4-methoxybenzophenone-5-sulfonic acid (BP-4), which serves as an ultraviolet filter, can enhance electron transfer and passivate interfacial defects, thereby improving the UV stability of perovskite devices by suppressing perovskite degradation. Similarly, by precisely tuning the concentration of 2-(2-hydroxy-5-methylphenyl)benzotriazole (UVP) additive in perovskite precursor, Li et al. [34] achieved an improvement in the overall performance of the perovskite absorption layer, exhibiting larger grain sizes, longer carrier lifetimes, and enhanced diffusion lengths. The current UV-absorbing Lewis base additives, such as BP‑4 and UVP, exhibit outstanding performance in promoting defect passivation and crystallization modulation. At the same time, they mainly act as passive UV filters and lack intentional molecular design to maximize dipole moments or utilize dynamic processes like tautomerization to boost passivation efficiency. Furthermore, the physical mechanisms that govern their interaction with perovskite interfaces remain unclear, and a comprehensive theoretical framework is still absent for designing high-performance UV-absorbing passivators. On the other hand, small organic Lewis bases with high dipole moments are known to improve defect passivation efficacy through stronger electrostatic interactions with charged perovskite surfaces, which reinforce chemical coordination and promote anchoring at defect sites [37–40]. Therefore, rational molecular design by tailoring dipole moments and introducing multifunctional anchoring sites (such as the –C=N electron-donating and proton-donating groups) can be an alternative strategy for maximizing defect passivation efficiency.

In this work, UV320 and UV327 molecules, a class of phenol triazine-based absorbers that are distinct from previously reported ones such as benzophenone BP‑4 and benzotriazole UVP, are introduced to enhance the stability and performance of perovskite photoelectronic devices. Their unique design, incorporating an electron‑withdrawing chlorine atom in UV327 and proximate –C=N and –OH groups, facilitates a keto–enol tautomerization under UV irradiation or heating. This process, combined with the dipole‑modulating effect of the substitutional chlorine atom, significantly increases the molecular dipole moment and dynamically enhances passivation at the perovskite/SnO_2_ interface, representing a mechanism previously unreported for this class of additives. Furthermore, UV327 regulates spatial compatibility through Cl–O bond interactions with perovskite lattices. Consequently, UV327-treated devices achieve superior performance: responsivity of 0.3 A W^−1^ and detectivity of 3 × 10^13^ Jones. Our work provides an effective approach for developing a multifunctional UV-resistant passivator.

## Results and Discussion

### Passivation Mechanism of UV-320 and UV-327 Molecules

**Fig. 1:**
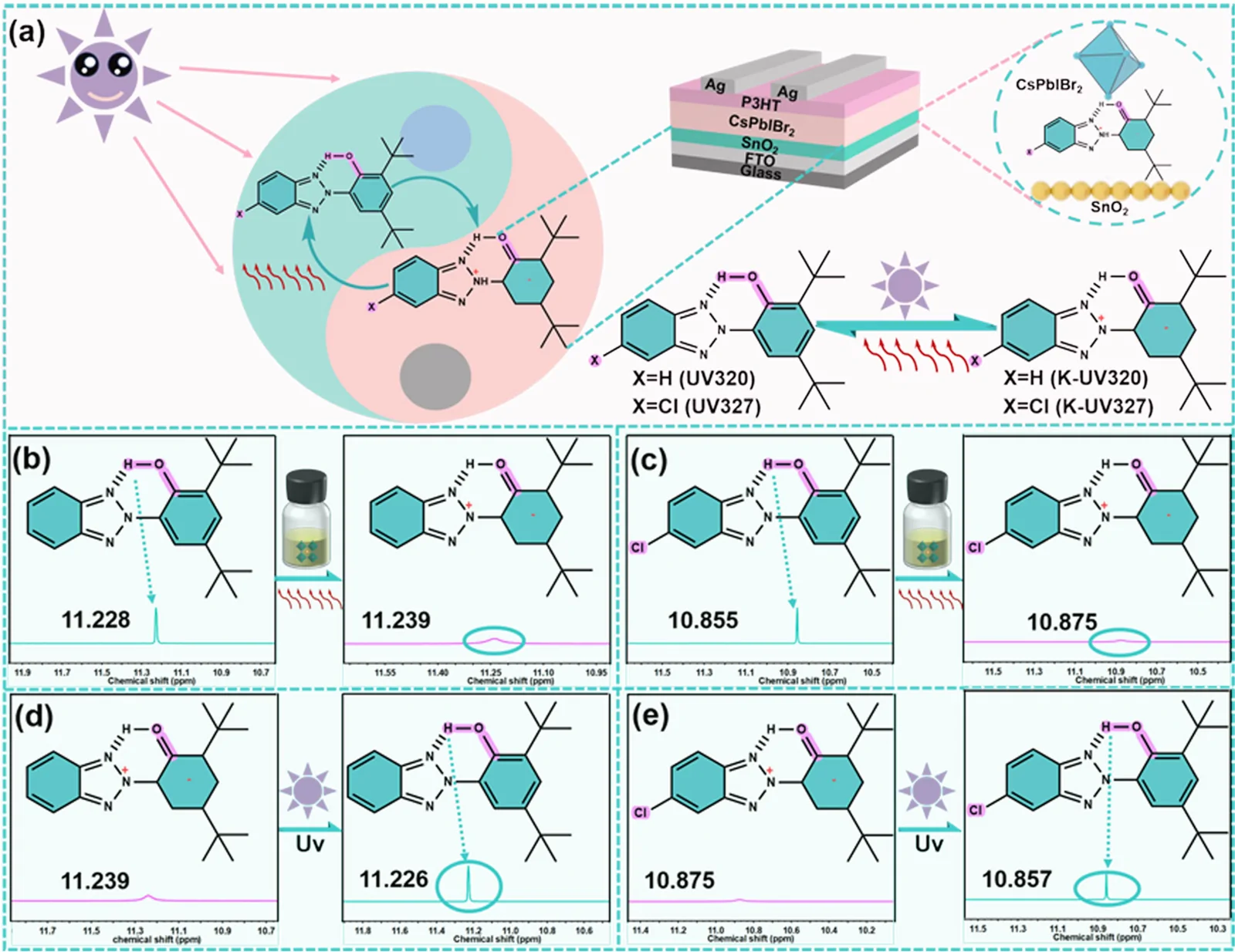
Passivation mechanism of UV320 and UV327 molecules. **a** A diagram illustrating the effect of UV320 and UV327 molecules on ultraviolet protection for perovskite. **b–c** ^1^H NMR spectra of UV320 and UV327 molecules before and after being added to the perovskite upon heating, respectively. **d–e** ^1^H NMR spectra of K-UV320 and K-UV327 molecules before and after being added to perovskite upon ultraviolet radiation, respectively

**Fig. 2:**
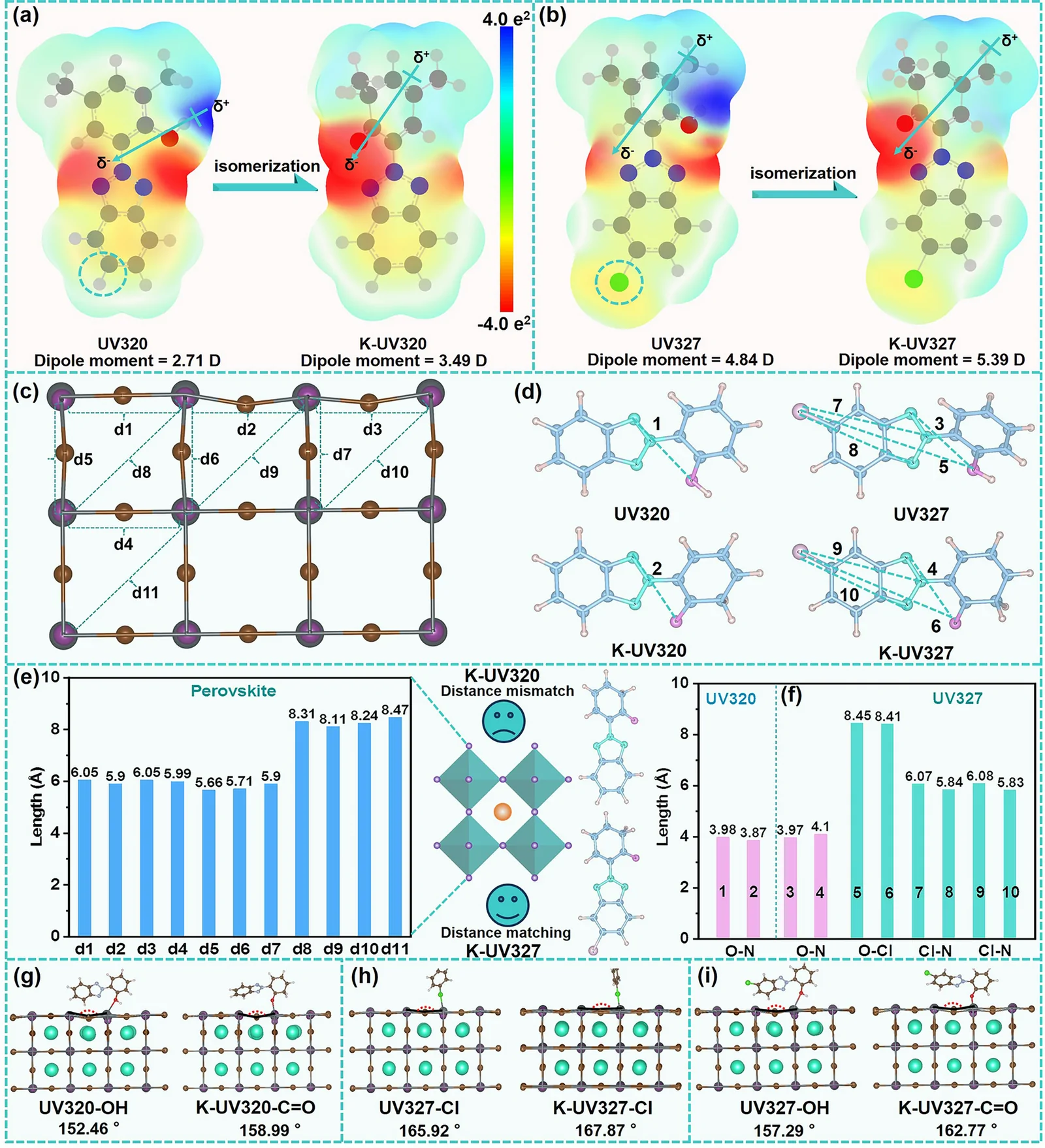
Calculation of UV327 and UV320 molecular electron cloud density distributions, as well as the calculation of bond lengths and bond angles. **a–b** Molecular electrostatic potentials of UV320 and UV327, as well as the keto form structures. **c** Spatial distance diagram of adjacent Pb^2+^ ions in perovskite. **d** Spatial distance diagram of N–N, N–O, and N–Cl atoms in both molecules. **e** Statistical chart of the spatial distance of adjacent Pb^2+^ ions in perovskite. **f** Statistical chart of the spatial distance of N–N, N–O, and N–Cl atoms in both molecules. **g–i** Bond angle size of Pb-Br-Pb after adsorption of functional groups in two molecules and their keto-form molecules with perovskite

In the work, UV327, UV320, BP-4, and UVP share a critical structural feature: -OH on their benzene rings that enable ultraviolet (UV) absorption. Figure 1a schematically illustrates the general mechanism by which these UV absorbers regulate perovskite properties. When incorporated into perovskite precursors or electron transport layers and subjected to heat or UV irradiation, the additives undergo excited-state intramolecular proton transfer (ESIPT), converting to keto forms with –C=O groups. Density functional theory (DFT) calculations reveal that the –C=O groups exhibit higher electron cloud density than their precursor –OH groups, indicating superior defect passivation capabilities post-tautomerization. To further confirm that the tautomerization-generated –C=O group has a stronger coordination ability toward Pb^2+^ than the original –OH group, Bader charge analysis on the oxygen atoms of both functional groups was performed after the adsorption of UV320, UV327, K-UV320, and K-UV327 molecules onto the perovskite. As shown in Fig. S1, the Bader charge transfer for the oxygen atoms in the –OH groups of UV320 and UV327 is 0.86*e* and 0.80*e*, respectively. Upon conversion to the –C=O group, the charge transfer increases to 0.96*e* and 0.98*e*, respectively. This observation is consistent with these calculations of molecular electrostatic potential (Fig. 2a, b), which present a higher electron density around the –C=O group than that around the -OH group, rationalizing its stronger coordination interaction capability. Furthermore, introducing electron-withdrawing groups enhances ESIPT efficiency under external stimuli (e.g., UV irradiation, heating), increasing the population of high-electron-density –C=O groups and thereby elevating molecular dipole moments. To leverage this effect, a chlorine atom (–Cl) was strategically introduced on the opposite benzene ring side of UV327 relative to UV320.

The nuclear magnetic resonance hydrogen (^1^H NMR) spectrometer was employed to verify the tautomerism process. For both UV320 and UV327 molecules, the comparative analysis of the ^1^H NMR spectra (Figs. 1b, c and S2–S3) reveals a significant decrease in the signal intensity of their -OH group after the two molecules were mixed into the perovskite precursor and heated at 60 °C for three h, respectively. Still, it remains detectable, showing low-field shifts of 0.011 and 0.02 ppm, respectively. This behavior results from the tautomerization of -OH groups to –C=O groups. Furthermore, the larger chemical shift seen in the UV327 molecule indicates a stronger chemical interaction with perovskite compared to the UV320 molecule. To evaluate the ultraviolet light absorption capability of the keto forms transformed from their corresponding UV320 and UV327 molecules and verify the reversibility of the tautomerism process, the ultraviolet lights were used to irradiate the thermal-treated perovskite precursor solution, and ^1^H NMR spectroscopy was employed to examine the –OH hydrogen in both molecules (Figs. 1d, e and S2–S5). These ^1^H NMR experiments indicate that UV320 and UV327 revert to their original phenolic forms after UV light irradiation. This conclusion is drawn from the quantification of their enolic and ketonic forms (Figs. S6 and S7), with the influence of DMSO-*d₆* on the equilibrium process also attached. Furthermore, the larger chemical shifts observed in UV327 compared to UV320 suggest that the introduction of the Cl atom promotes the ESIPT process, consistent with the chemical shift results mentioned above.

To elucidate the evolution of charge distribution during the tautomeric transformations from the keto forms of UV320 and UV327 molecules to those of K-UV320 and K-UV327, respectively, following their (named), the molecular electrostatic potentials of each compound were calculated individually based on DFT. As shown in Figs. 2a, b and S8, the intramolecular electron cloud (highlighted in red) in UV320 and UV327 molecules is primarily localized around the –C=N, –OH, and –Cl group sites. These functional groups serve as effective Lewis bases, facilitating the donation of lone-pair electrons to undercoordinated Pb^2+^ (V_I_) in perovskite [35, 36, 38, 41]. Critically, the superior performance of UV327 over UV320 can be attributed to its unique Cl atom. Functionally, this atom serves as a potent electron-withdrawing group, which enhances the molecular dipole moment, thereby facilitating more effective electrostatic anchoring of defects. Upon the tautomerization of UV320 and UV327 molecules into their keto forms, electrons are predominantly localized around the –C = N and –C = O groups, with a notably intensified coloration, indicating a stronger electron-donating capacity of the –C = O group compared to the –OH functional group. The enhanced electron-donating ability allows the –C = O group, with its high electron cloud density, to effectively passivate undercoordinated Pb^2+^. Additionally, UV327 (μ = 4.84 D) and K-UV327 (μ = 5.39 D) exhibit greater molecular dipole moments than UV320 (μ = 2.71 D) and K-UV320 (μ = 3.49 D), enhancing their ability to fix positively charged ions like Pb^2+^ within the PbX_6_^4−^ octahedral perovskite structure [37, 42]. According to the DFT calculations on the adjacent Pb-Pb atomic separation in the perovskite and N–O, Cl–O, and Cl–N bond lengths in the above-mentioned four molecules, as shown in Fig. 2c, d, and the statistical results shown in Fig. 2e, f. Beyond its electronic effects, the Cl atom in UV327 also exhibits the ability to further optimize the molecular spatial configuration. The spatial distances of the Cl-N bonds in UV327 and K-UV327, along with the O-Cl bonds, exhibit a more matched alignment with the spatial distances of the two adjacent Pb atoms in the perovskite lattice compared to the N–O bond length in UV320 and K-UV320, as evidenced by the significant increase of the comprehensive spatial matching score from ~ 0.7 to 0.9 [41]. This natural merit in the molecular configuration endows UV327 and K-UV327 with stronger passivation efficiency than UV320 and K-UV320. The Pb-Br-Pb bond angles influenced by the different functional groups in the four molecules adsorbed upon the perovskite lattice were also calculated, as shown in Fig. 2g, i. The observed larger Pb-Br-Pb bond angles suggest that the UV327 molecule and its keto-form counterpart minimize the lattice distortion and surface stress, meanwhile improving the structural stability [43, 44]. In summary, the Cl atom serves a multifaceted role: It enhances the molecular dipole for electrostatic anchoring, promotes tautomerism to activate stronger Lewis basic sites, and optimizes spatial compatibility for reduced interfacial strain, collectively contributing to superior multi-site defect passivation, improved charge transport, and enhanced device stability.

Time-of-flight secondary ion mass spectrometry (TOF–SIMS) was employed to determine whether Cl atoms incorporate into the perovskite surface or lattice. The TOF–SIMS mass spectrum, depth profile, and 3D rendering confirm the presence of UV327 (Figs. S9 and S10). A pronounced Cl^−^ signal at the film surface demonstrates its successful incorporation at the perovskite interface. In addition, the ubiquitous distribution of the Cl^−^ signal across the perovskite layer indicates deep diffusion of UV327, leading to enhanced bulk passivation. Consequently, UV327 possesses dual function: passivating surface defects and suppressing deep-level traps distributed in the entire film, both can reduce the defect density effectively.

### Impact of UV-320 and UV-327 Molecules on Crystallinity and Film Quality of Perovskite

**Fig. 3:**
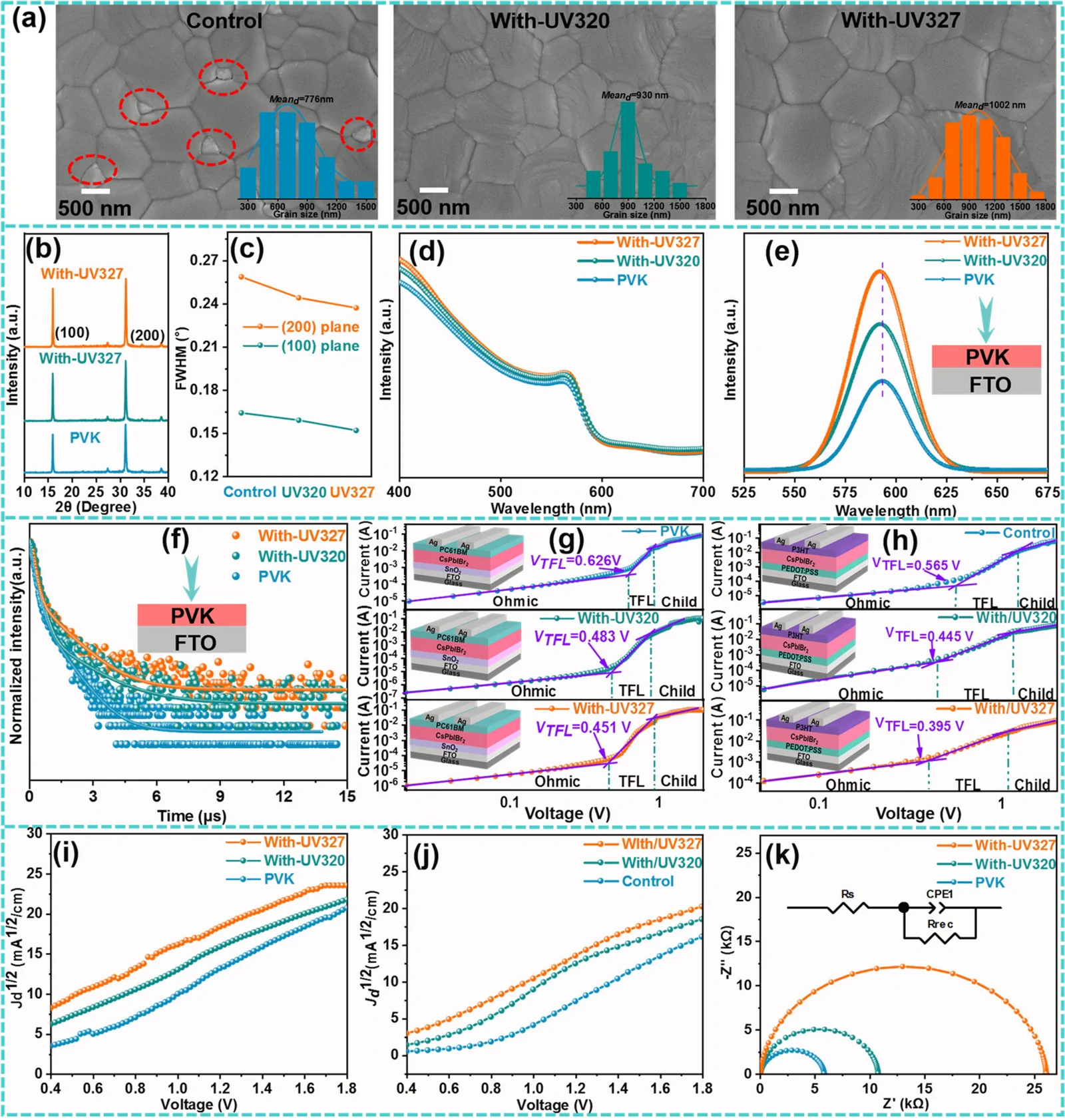
Characterization of perovskite film quality. **a** SEM images, **b** XRD patterns, **c** FWHM values of the (100) and (200) diffraction peaks, **d** UV–Vis absorption spectra, **e** steady-state PL spectra, and **f** time-resolved photoluminescence (TRPL) spectra of perovskite films with and without UV320 or UV327 treatment. Current–voltage (*I-V*) characteristics of (**g, i**) electron-only and (**h, j**) hole-only devices based on pristine, UV320-, and UV327-treated perovskite films. **k** Nyquist plots of the devices based on perovskite films with and without UV320 or UV327 treatment

The influence of UV320 and UV327 molecules on the crystallinity of perovskite films was systematically investigated using X-ray diffraction (XRD). As shown in Fig. 3b, c, the modified perovskite films exhibit stronger XRD intensities and narrower full width at half maximum (FWHM) values compared to the control, indicating enhanced crystallinity and improved preferred orientation [39, 45, 46]. This suggests that both molecules likely facilitate a more ordered crystal growth, probably by passivating surface defects and reducing nucleation barriers during film formation [47, 48]. Among them, UV327 demonstrates the optimal passivation effect. Scanning electron microscopy (SEM) images reveal substantial morphological improvements in the treated perovskite films. Relative to the control film (776 nm), the average grain size increased progressively with UV320 (930 nm) and UV327 (1002 nm) treatments, as quantified in Fig. 3a. Consequently, the UV327-modified film not only possesses the largest grains but also exhibits a superior polycrystalline morphology characterized by fewer pinholes and a smoother surface. These observations are further supported by atomic force microscopy (AFM) results (Fig. S11), which show a reduction in RMS roughness from 40.32 nm (control) to 31.00 nm (UV320-treated) and 28.66 nm (UV327-treated). The surface smoothing can be attributed to the reduction of the energy barrier for grain coalescence and promotion of secondary crystallization induced by passivation effects [37, 47].

The optical performance of the perovskite films was also optimized upon modification. UV–visible absorption spectra (Figs. 3d and S12–S13) show that all films maintain the same bandgap (2.07 eV). However, the UV320- and UV327-modified samples exhibit marginally stronger absorption at shorter wavelengths, which likely stems from enhanced light scattering due to larger grain sizes and improved film compactness [45]. Steady-state photoluminescence (PL) and time-resolved photoluminescence (TRPL) spectra were obtained to further investigate the effects of the two molecules on the quality of the perovskite film and carrier transport behaviors. Compared to the PL peak (Fig. 3e) position of the control sample at 590 nm, the PL peaks of both treated samples undergo slight blue shifts [41]. Additionally, the UV327-treated sample exhibits the most intense PL under the normalized excited laser power, implying more effective suppression of non-radiative recombination benefited from better defect passivation at grain boundaries and interfaces [4, 39, 41, 45]. The TRPL decay curves (Fig. 3f) were well fitted by using a double-exponential function (Eq. S1), and the fitted data are summarized in Table S1. The average PL lifetime (*τ*_*ave*_) of pristine perovskite film is 370.76 ns, while those of UV320 and UV327-treated samples are 460.64 and 470.71 ns, respectively. The UV327-treated sample exhibits a 1.27-fold extension in carrier lifetime than that of the control one, suggesting a reduction in non-radiative recombination centers, which is consistent with the observed improvements in crystallinity and interfacial quality [1, 39, 41]. To quantitatively assess the defect density of states within the UV320 or UV327-treated perovskite film, the space-charge limited currents (SCLC) method with devices of electronic-only and hole-only was well conducted. The trap density *N*_*d*_ can be given by Eq. 1 [41]:

1 $${N}_{d}=\frac{2{\varepsilon }_{0}\varepsilon {V}_{\mathrm{TFL}}}{e{L}^{2}}$$

where *V*_TFL_, *e*, *N*_*d*_, *L*, *ε,* and *ε*_0_ are the trap-filled limit voltage, elementary charge, trap density, perovskite film thickness, relative permittivity of the perovskite, and vacuum permittivity, respectively. Electron-only devices with an FTO/PEDOT: PSS/perovskite/P3HT/Ag structure were fabricated to measure dark current–voltage characteristics. Results show that UV327-modified devices exhibit a lower *V*_*TFL*_ (Fig. 3g) [11, 41]. According to the calculations, the electron trap state densities of pristine and treated devices UV320 and UV327 are 6.12 × 10^15^, 4.72 × 10^15^, and 4.41 × 10^15^ cm^−3^, respectively. Similarly, the hole-only devices with FTO/SnO_2_/ Perovskite/PCBM/Ag structure were fabricated to confirm the hole trap state densities (see Fig. 3h). The calculated *N*_*d*_ values of the perovskite in control and target groups are determined to be 5.52 × 10^15^, 4.35 × 10^15^, and 3.86 × 10^15^ cm^−3^, respectively. The decrease behavior of trap state density, which is confirmed by the reduced dark current as shown in Fig. 6a, results from the effective passivation of both ionic and vacancy-related defects at grain boundaries and within the bulk [11, 14].

Additionally, increased electron and hole mobilities (Fig. 3i, j) [11, 49] and enhanced recombination resistance derived from Nyquist plots (Fig. 3k) [37, 40] collectively confirm that both UV320 and UV327 contribute to improved charge transport and reduced defect-mediated recombination, with UV327 exhibiting superior passivation performance.

### Impact of UV-320 and UV-327 Molecules on the Morphology and Band Structure of Perovskite

**Fig. 4:**
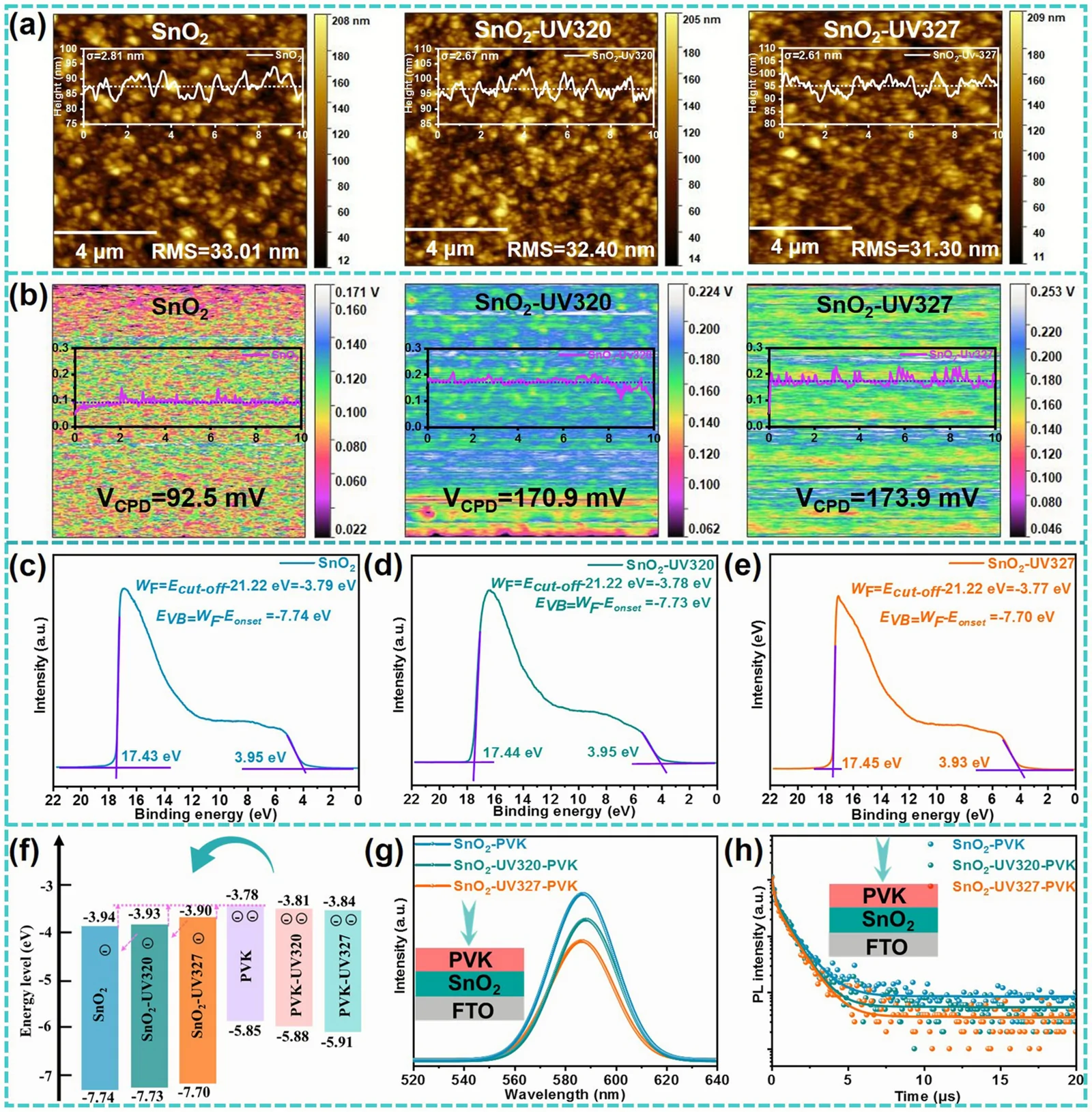
Effects of UV320 and UV327 molecules on the morphology and electronic properties of SnO_2_. **a** AFM and **b** KPFM images of SnO_2_ and the modified SnO_2_ film with UV320 and UV327. UPS spectra of **c** SnO_2_, **d** SnO_2_-UV320, and **e** SnO_2_-UV327 films. **f** Schematic diagram of changes in the band structure of SnO_2_ by two molecular modifications. **g** Steady-state PL spectra, and **h** TRPL spectra of the perovskite film with and without UV320 and UV327 treatment

An important aspect of our study is the investigation into the effects of UV320 and UV327 molecules on the SnO_2_ electron transport layer (ETL), beyond their interaction with the perovskite. AFM characterization shows that the RMS roughness of SnO_2_ films decreases from 33.01 nm (control) to 32.40 nm (with UV320) and 31.30 nm (with UV327) (Figs. 4a and S14). The passivation of undercoordinated Sn sites by the Lewis basic groups (e.g., C = 0) of the UV320 and V327 molecules results in this smoothing process occurring on the SnO_2_ ETL surface. This coordination reduces surface energy heterogeneity and nanoscale irregularities, thereby promoting a more uniform substrate for subsequent perovskite deposition [50, 51]. Furthermore, Kelvin probe force microscopy (KPFM) was employed to investigate the influence of UV320 and UV327 on the surface contact potential difference (*V*_*CPD*_). Figures 4b and S15 show that the average *V*_*CPD*_ of pristine SnO_2_ films increased from 92.5 mV to 170.9 and 173.9 mV after treatment with UV320 and UV327, respectively. The observed *V*_*CPD*_ increase suggests a shift in surface potential, likely induced by molecular dipole moments of UV320/UV327, which modify the local electronic environment of SnO_2_. As reported in previous studies, an elevated *V*_*CPD*_ leads to an upward shift of the conduction band minimum (CBM) of SnO_2_ [51]. This realignment reduces the energy barrier at the SnO_2_/perovskite interface, thereby promoting electron injection and suppressing non-radiative recombination [51, 52].

To verify the above conclusion, ultraviolet photoelectron spectroscopy (UPS) was employed to determine the valence band maximum (VBM) of the SnO_2_ and perovskite films, as shown in Figs. 4c–e and S17, as well as a corresponding energy level diagram illustrated in Fig. 4f. The CBM/VBM of the pristine perovskite is located at − 3.78 and − 5.85 eV, respectively. Upon modification with UV320 and UV327, the corresponding values shift to − 3.81 and − 5.88 eV, respectively, and to − 3.84 and − 5.91 eV, respectively. Concurrently, the SnO_2_ ETL modified with UV320 and UV327 exhibits CBM values of − 3.93 and − 3.91 eV, respectively, compared to − 3.94 eV for the pristine one. This collective band realignment results in a more favorable energy level matching at the interface, effectively reducing the electron injection barrier. The observed shifts in the SnO_2_ CBM are primarily attributed to adsorbate passivation of surface defects. In the case of UV327, the electronegative chlorine atom also contributes to its overall passivation effect, since Cl atoms can interact with under-coordinated Sn atoms and oxygen vacancies. Therefore, the combined effect of these factors modifies the surface electronic state and grain boundary potential without changing the crystal sizes of the SnO_2_ film. This modification subsequently facilitates carrier transport and extraction within the corresponding PDs. This passivation effect is corroborated by the suppressed PL intensity (Fig. 4e) and reduced carrier lifetime (Fig. 4f, Table S2), confirming the suppression of non-radiative recombination and the consequent facilitation of carrier transport and extraction in the corresponding devices.

### Interaction between UV-320 and UV-327 Molecules and Perovskite

**Fig. 5:**
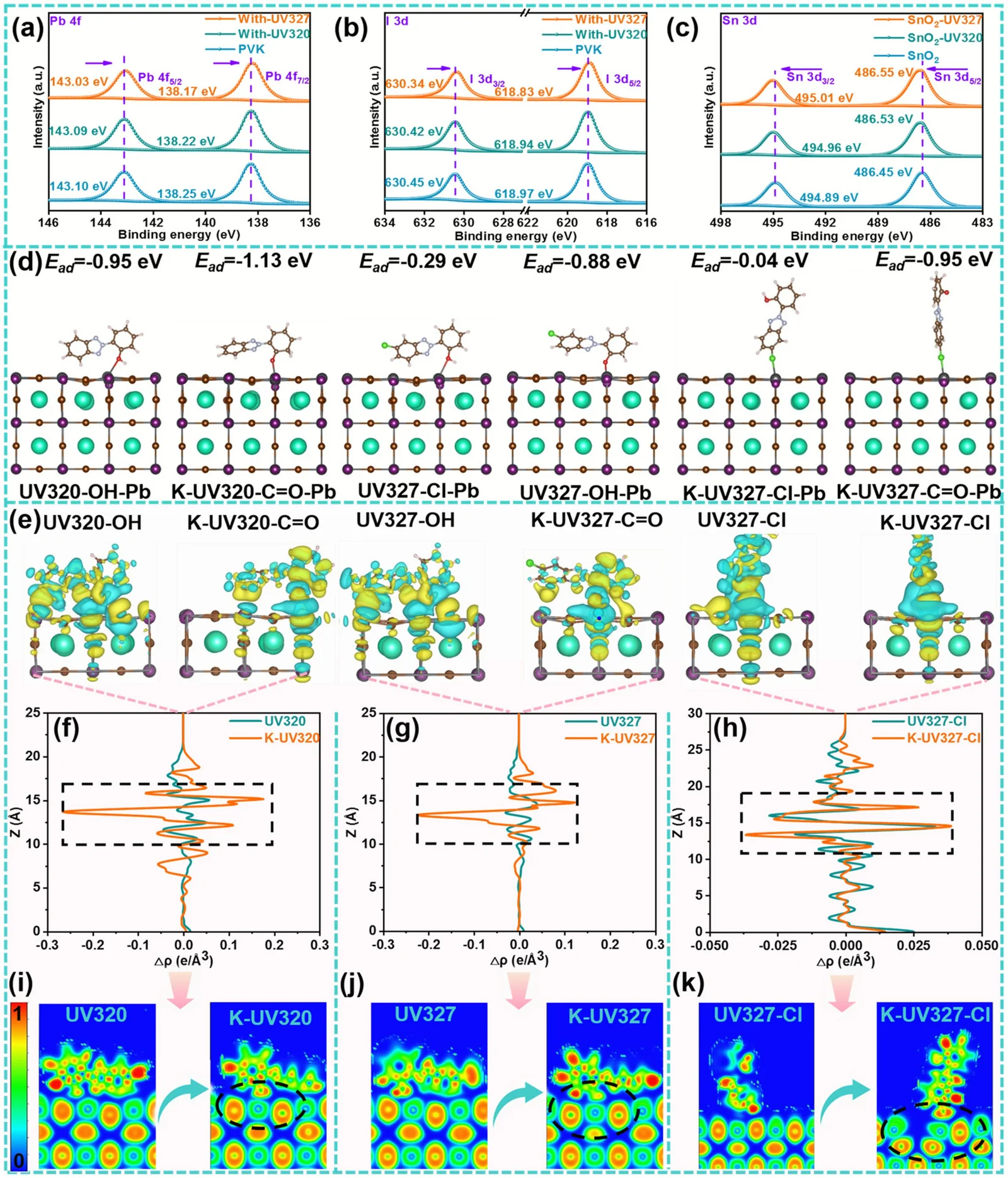
XPS spectra of **a** Pb 4*f* and **b** I 3*d* for PVK, UV320-PVK, and UV320-PVK films; **c** Sn 3*d* for SnO_2_, UV320-SnO_2_, and UV327-SnO_2_ films. **d** Adsorption models of different functional groups in UV320 and UV327 with perovskite, along with the statistical results of the adsorption energy (Note: The adsorption strength increases with the magnitude of the negative value). **e** Charge density difference of K-UV320 and K-UV327 after adsorption of the perovskite. The charge displacement profiles of **f** –C=O groups in UV320 and K-UV320, **g** –C=O groups in UV327 and K-UV327, and **h** Cl atom in UV327-Cl and K-UV327-Cl, with the perovskite adsorption. The electron localization function **i** –C=O groups in UV320 and K-UV320, **j** –C=O groups in UV327 and K-UV327, and **k** Cl atom in UV327-Cl and K-UV327-Cl, with the perovskite adsorption

The interfacial interaction mechanisms between the modified molecule and perovskite or SnO_2_ were also investigated based on X-ray photoelectron spectroscopy (XPS) analysis. As shown in Fig. 5a, all samples exhibit similar characteristic peaks of Pb 4*f* XPS spectra. After modification by UV320 and UV327, the Pb 4*f*_5/2_ and Pb 4*f*_7/2_ characteristic peaks shift to lower binding energies by 0.01, 0.07, 0.03, and 0.08 eV, respectively. However, a similar phenomenon is also observed in the I 3*d* XPS (Fig. 5b) and Cs 3*d* and Br 3*d* characteristic peaks (Fig. S17); the characteristic peaks of I 3*d*_3/2_ and I 3*d*_5/2_ shift to lower binding energies by 0.03, 0.21, 0.03, and 0.14 eV, respectively. The decreasing binding energies of the I 3*d* and Pb 4*f* orbitals in the perovskite films suggest that the functional groups –C = O, –C = N, and –Cl atoms in the UV320 and UV327 molecules effectively coordinate with free I^−^ and uncoordinated Pb^2+^ [53]. Therein, the UV327 molecule exhibits a more significant effect in curing defects related to perovskite, including reducing the hysteresis and I^−^ migration and oxidation, thereby enhancing the stability of the PDs [11, 53]. As shown in Fig. 5c, the Sn 3*d*_3/2_ and Sn 3*d*_5/2_ shift to higher binding energies by 0.07, 0.12, 0.08, and 0.10 eV after the SnO_2_ film was treated by UV320 and UV327, respectively. The increasing binding energy of Sn 3*d* indicates an interaction and strong electron transfer between SnO_2_ and the UV320 and UV327 molecules interlayer, which reduces the electron cloud density of Sn [54]. This conclusion is further substantiated by the increased lattice oxygen ratio in the SnO_2_ film (Fig. S18) and the distinct chemical shifts in Sn 3*d* XPS spectra. The Sn 3*d* binding energy shift for UV320 is primarily attributed to coordination from its –C=O group (Fig. S19c). In contrast, UV327 induces a more pronounced shift due to the synergistic coordination of its multiple functional groups (–C=N, –C=O, and the electron-withdrawing –Cl atom), as evidenced in Figs. S19a, b and S19d, e. More importantly, the Cl atom plays a pivotal role in promoting keto–enol tautomerism and enhancing the Lewis basicity of the O and N coordinating atoms. This electronic enhancement enables UV327 to provide superior defect passivation over UV320, effectively suppressing non-radiative recombination and improving device stability. Fourier transform infrared spectroscopy (FTIR) was further employed to probe the interactions among the additive molecules, perovskite, and SnO_2_. The stretching vibrations of the –C=N and –C=O functional groups in UV327, which are observed at 1599.0 and 1631.4 cm^−1^ after heating, exhibit noticeable shifts (Fig. S20). These shifts occurred in UV327, which are more pronounced than those observed in UV320 and are similar to the behavior in UVP molecules, indicating stronger chemical coordination between the -C=O group of UV327 and the perovskite/SnO_2_ interface [4].

To understand the effects of functional molecules UV320 and UV327 and their tautomer on the defect passivation of perovskite, the DFT approach was employed to calculate the chemical interactions between the functional groups within these two molecules and the CsPbIBr_2_ perovskite with the V_I_ defect-terminated. Therein, the corresponding adsorption models and statistical results of adsorption energy are presented in Fig. 5d. For the UV320 and UV327 molecules that have not undergone tautomerism, the adsorption energy (*E*_*ad*_) of the -OH and Cl atoms in UV327 for Pb^2+^ is − 0.29 and − 0.88 eV, respectively, and their synergistic passivation effect is stronger than that of the -OH functional group in UV320 (*E*_*ad *_= −0.95 eV). After the tautomerism from their primitive structures to keto counterparts, the *E*_*ad*_ of the –C = O coupling with Pb^2+^ in both molecules is higher than that of the original –OH groups; this calculated result is well in agreement with the previous ESP and dipole moment results [4]. The relatively lower *E*_*ad*_ value of the –C=O in UV320 probably can be ascribed to the strong electron-withdrawing effect of the –Cl atom, with which some electrons confined in the –C=O group are expropriated, and did not consider the functional group with a high electron density of the –C=N. However, the overall synergistic effect of defect passivation by UV327 is superior to that of UV320.

The charge density difference (CDD) between the two molecules and the defective perovskite was calculated, respectively, to further investigate the charge transfer at the SnO_2_/perovskite interface. The adsorption models of CDD and the corresponding extracted values of interfacial charge densities along the z-axis, which is perpendicular to the SnO_2_/perovskite interface, are illustrated in Fig. 5e and 5f–h, respectively. During the formation of chemical bonds, charge rearrangement occurred in adjacent regions, accompanied by strong charge transfer, thereby substantiating the interaction of both molecules with the perovskite surface [11, 55]. Notably, both the keto-form structures of UV320 and UV327 exhibit distinct charge transfer properties on the SnO_2_/perovskite interface, which is quite different from their parent structure’s isomerization. Furthermore, the Cl atom introduced into the UV327 molecule exhibits a similar behavior at the perovskite contact interface (Fig. 5h). Therefore, the keto-form structures of both UV320 and UV327 also have superior effectiveness in passivating perovskite film defects and suppressing ion migration. Furthermore, the strategic introduction of electron-withdrawing groups to the aromatic ring can significantly enhance these passivation effects. While a slightly stronger charge transfer is demonstrated between K-UV320 and perovskite than that between K-UV327 and perovskite, since the electron-withdrawing chlorine atom in the UV327 molecule triggers the delocalization of electron density into the carbonyl group. This charge transfer mechanism precisely explains why the adsorption energy between the –C=O group in K-UV320 and perovskite is slightly lower than that in K-UV327. Notably, while the chlorine atom incorporated in K-UV327 marginally reduces the adsorption capability of the –C=O group, this process remarkably optimizes the interfacial geometry of perovskite. Specifically, K-UV327 possesses more favorable intermolecular spacing and optimized bonding angles, which synergistically contribute to its superior defect passivation and interface modification performance compared to K-UV320. These conclusions are further corroborated by electron localization function (ELF) analysis (Fig. 5i–k), which shows more pronounced interfacial electron density redistribution in K-UV327 [56]. This enhanced charge delocalization facilitates the formation of robust Pb–O bonds on the perovskite surface.

### Influence of UV-320 and UV-327 Molecules on Device Performance

**Fig. 6:**
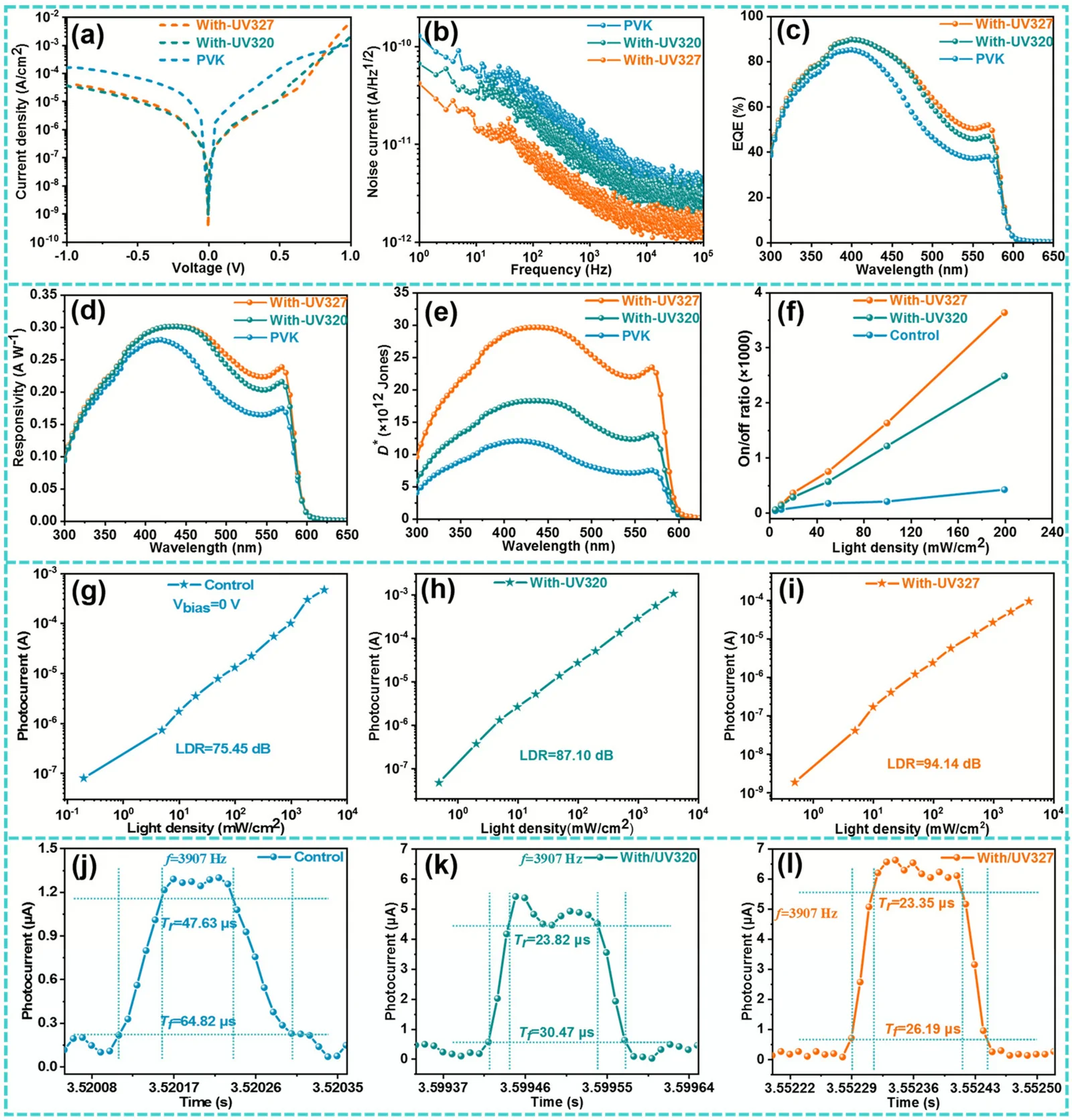
Photoelectronic performance of PPD devices modified with UV320 and UV327. **a** Dark *J-V* curves of PPDs. **b** Noise current of PPD devices with and without UV320 and UV327 treatment. **c** *EQE* curves. **d** *Responsivity* (*R*). **e** Detectivity (D*). **f** On/off ratios under various optical power densities. The linear dynamic range (LDR) of **g** control of PPDs, **h** PPDs treated by UV320, and **i** PPDs treated by UV327. The performance of response time of **j** control PPD devices, **k** PPDs treated by UV320, and **l** PPDs treated by UV327

The photosensitive properties of the perovskite photodetector (PPD) devices with the structure FTO/SnO_2_ (UV320 or UV327)/perovskite/P3HT/Ag were investigated. For clarity in subsequent discussions, the devices were designated as (unmodified), PD320 (UV320-modified), and PD327 (UV327-modified). In Fig. 6a, PD327 exhibits a dark current density (I_d_) of 3.22 × 10^−10 ^A cm^−2^, which is approximately 2.6 times lower than that of PD320 (8.50 × 10^−10 ^A cm^−2^) and about 5 times lower than that of PD0 (1.69 × 10^−9 ^A cm^−2^). The significant reduction of *I*_*d*_ indicates markedly suppressed leakage current, which originates from two key modifications induced by UV327. Firstly, the improved morphology of the SnO_2_ film eliminates pinholes in ETL, blocking a major physical shunt pathway; meanwhile, the optimized energy level alignment raises the electron injection barrier under reverse bias, effectively suppressing the injected current density. Secondly, within the perovskite layer, the effective passivation of bulk and interface by UV327 significantly decreases the density of trap states [15, 19], which are the primary sources for the trap-assisted generation current (Shockley–Read–Hall recombination).

This comprehensive suppression of leakage pathways is consistent with the large XPS spectral shift, enhanced charge transfer resistance (Fig. 3k), and lower noise current (Fig. 6b) observed in PD327. External quantum efficiency (*EQE*) is a crucial performance metric for photodetectors, defined as the ratio of the number of electrons collected by the photodetector to the total number of incident photons. The *EQE* response across the wavelength range of 300 to 600 nm is shown in Fig. 6c. The maximum *EQE* values for PD0, PD320, and PD327 are 85.14, 89.71, and 89.75%, respectively. The responsivity (*R*) is defined as the ratio of the output photocurrent to the incident optical power and can be expressed as [16]: $R=\frac{EQE\lambda q}{hc}$, where λ represents the wavelength, *q* denotes the elementary charge, *h* signifies the reduced Planck constant, and *c* is the speed of light in a vacuum. Since the *EQE* of PD327 surpasses that of PD0 and PD320, the *R*-value of PD327 (0.301 A W^−1^) is consequently greater than that of PD0 (0.280 A W^−1^) and PD320 (0.300 A W^−1^), as shown in Fig. 6c–d. The detectivity (D*) indicates the capability of photodetectors (PDs) to detect weak signals. When the noise in the PDs primarily arises from grain noise, D* can be expressed as [14, 16]: D* = *R* $\sqrt{{\mathrm{A}}\mathrm{/(}{\mathrm{2e}}{\mathrm{I}}_{\mathrm{d}}\mathrm{)}}$, where *e* is the elementary charge and *I*_*d*_ is the dark current. As shown in Fig. 6e, the D* of PD327 is consistently greater than that of PD0 and PD320, with a peak value of 2.96 × 10^13^ Jones, compared to 1.20 × 10^13^ Jones for PD0 and 1.82 × 10^13^ Jones for PD320 (1 Jones = 1 cm Hz^1/2^ W^−1^). The enhanced D* primarily results from improved responsivity and reduced dark current density, making them highly competitive among the recently reported all-inorganic PPDs (Table S3). In addition, PD327 and PD320 show 8.50 and 5.84 times higher on/off ratios (Fig. 6f) than those of PD0, respectively. This result can be attributed to more efficient charge extraction and reduced current leakage, mainly due to effective passivation of defects in CsPbIBr_2_ by both molecules [19, 57].

The linear dynamic range (*LDR*) indicates the range of light intensities within which the PD maintains constant responsivity, defined as: $\mathrm{LDR}=20 \mathrm{log}\frac{{I}_{\mathrm{upper}}}{{I}_{\mathrm{lower}}}$, where *I*_upper_ and *I*_lower_ represent the upper and lower limit of the light intensity maintaining linear photoresponse, respectively. As shown in Fig. 6g–i, all devices exhibit a linear response under 405 nm wavelength light. Through data fitting, the LDR of PD0 is 74.45 dB, while the LDR of PD320 and PD327 is 87.10 and 94.14 dB, respectively. This indicates that PD327 is more suitable for operation over a wider range of light intensities [19]. A critical parameter for photodetectors is the rise and fall times of the transient photocurrent. The rise time (*T*_rise_) refers to the time required for the photocurrent to increase from 10% to 90% of its maximum value, while the fall time (*T*_fall_) refers to the time required for the photocurrent to decrease from 90% to 10% of its maximum value [58]. As for the transient photocurrent, the significantly faster photocurrent response of PD327 (*T*_rise_/*T*_fall_ = 23.35/26.19 μs) compared to PD0 (47.63/67.82 μs) and PD320 (23.82/30.47 μs) (Fig. 6j–l) can be attributed to several synergistic factors rooted in the effective passivation and interface modulation induced by UV327. First, the reduced defect density and suppressed trap-assisted recombination, which result from the strong coordination of the –C = N, –C = O, and –Cl groups with undercoordinated Pb^2+^ and halide vacancies, facilitate quicker carrier transport and reduce trapping/detrapping dynamics. Second, the improved interfacial contact at the perovskite/SnO_2_ heterojunction and the optimized energy level alignment lower the energy barrier for charge injection, resulting in the improvement of carrier extraction efficiency. Together, these effects contribute to the enhanced response speed observed in PD327.

### Influence of UV-320 and UV-327 Molecules on the Stability of Perovskite Devices

**Fig. 7:**
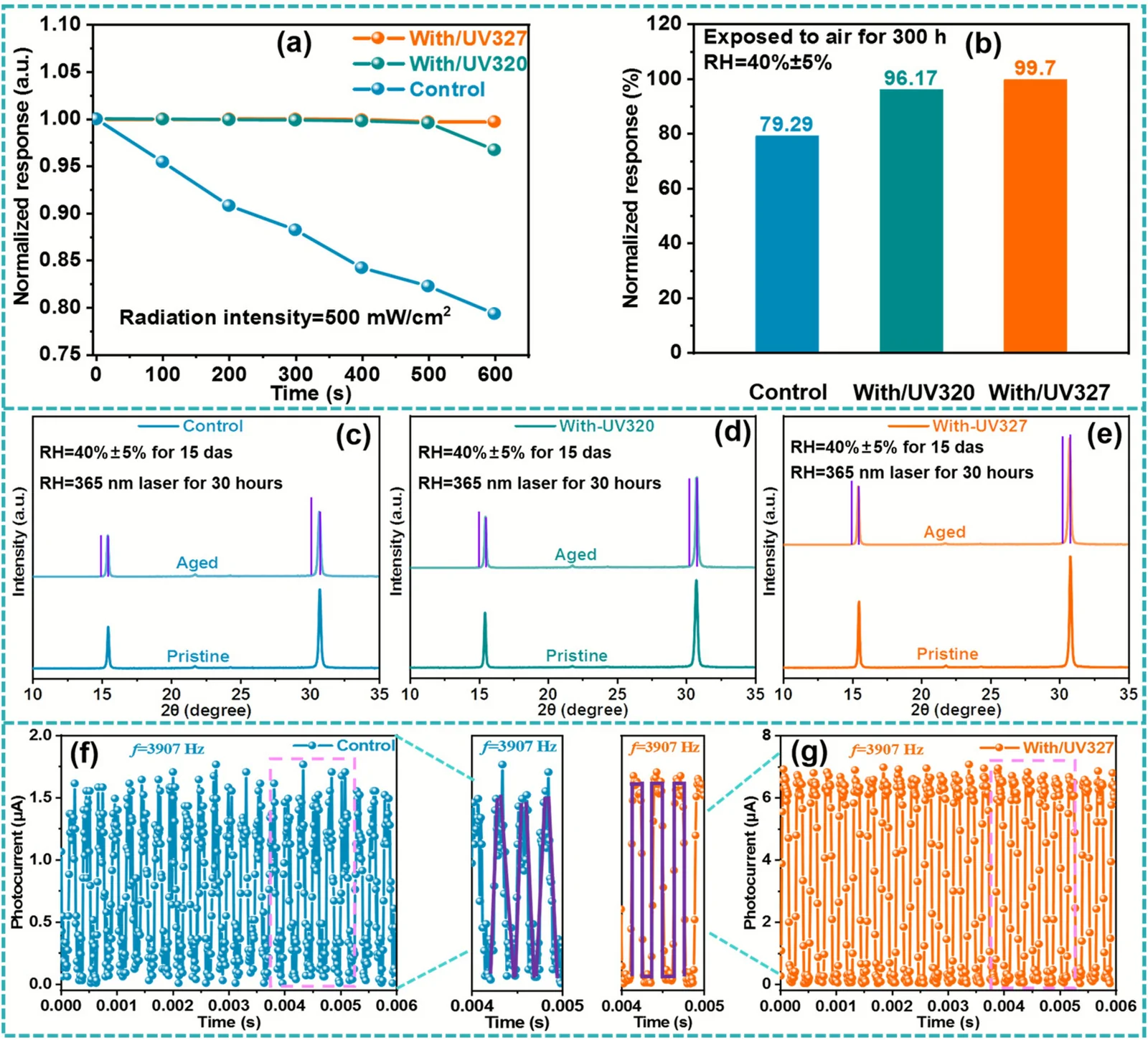
Photocurrent response and the stability of all devices. **a** Storage stability of unencapsulated PPD device under light illumination at 500 mW cm^−2^ with 40% ± 5% relative humidity. **b** Response change of all devices after 600 s *I-T* cyclic curve under 0 V bias voltage, 40% ± 5% relative humidity, and 500 mW cm^−2^ radiation intensity at 405 nm laser. XRD images of unencapsulated perovskite films are exposed to air for 15 days at a relative humidity of 40% ± 5% and subjected to 30 h of 365 nm laser radiation. **c** Pristine perovskite film, **d** with UV320, and **e** UV327. The photocurrent response curve of **f** PD0 and **g** PD327 at 3907 Hz

The unencapsulated devices were exposed to an environment with a humidity of 40% ± 5% for 300 h to test their stability (Fig. 7a, b). The *I-T* cyclic curve of PD327 and PD320 was measured under 0 V bias voltage for 600 s with a light intensity of 500 mW cm^−2^, and they maintained 99.70% and 96.17% of their initial photocurrent, respectively, compared with 79.29% for PD0. Additionally, three types of bare perovskite films were exposed to air for 15 days and then subjected to 30 h of UV irradiation at 365 nm. The decrease in peak intensity in the XRD patterns for the UV-320 and UV-327 modified films is significantly slower than that observed in the control group (Fig. 7c–e), robustly demonstrating the enhanced resistance to the erosion of humidity and UV light. Furthermore, the exceptional stability achieved by molecular modification suggests a high potential for improving thermal stability. This anticipated resilience stems from two key factors: (1) the intrinsic thermal robustness of the benzotriazole core, and (2) the effective suppression of ionic defects (particularly I^−^ vacancies and uncoordinated Pb^2+^) throughout the perovskite bulk, as confirmed by XPS and PL, TRPL and the dark current date which is expected to mitigate the ion migration that predominantly drives thermal degradation at elevated temperatures. Finally, the unencapsulated three devices were placed in the air for 300 h, and their *I-T* cyclic curve at 3907 Hz was subsequently measured, as shown in Figs. 7f, g and S21. PD327 exhibits better high-frequency response than the PD320 and PD0 devices, demonstrating superior signal response at relatively high frequencies.

In perspective, UV320 and UV327 constitute promising candidate additives for scaling perovskite photovoltaics. Their commercial availability, direct process compatibility with large-area coating techniques, and low required dosage result in negligible cost addition. This material cost is decisively offset by the concurrent improvement in device stability and efficiency, paving the way for the application of the two promising UV molecules in developing cost-effective and durable perovskite optoelectronic devices.

## Conclusions

In this work, two ultraviolet absorbers (UV320 and UV327) were chosen elaborately as the effective phenolic passivators of solution-treated halide perovskites. It is found that thermal-induced excited-state proton transfer can trigger the tautomerization of the two molecules from their intrinsic forms to their keto counterparts, i.e., K-UV320 and K-UV327, accompanied by the increasing quantity of –C=O groups in the tautomers. Moreover, the Cl- ion substituting –H bond site can further increase the dipole moment of the K-UV327 molecule and provide a more compatible spatial arrangement matched with the perovskite lattice, exhibiting stronger efficacy of chemical coordination, defect passivation, and trap-state elimination for perovskite and SnO_2_ layers than that of UV320. Furthermore, this modification strategy promotes the crystallization of perovskite and SnO_2_ films and then enhances the electron extraction capability in the multilayer film system. These optimizations achieved on materials create the superior performance of self-powered PPD: a peak *EQE* of 89.75% in the 300–600 nm range, a low dark current density of 3.22 × 10^–10^ A cm^−2^, a high responsivity of 0.301 A W^−1^, and a maximum specific detectivity of 2.96 × 10^13^ Jones. Furthermore, the devices modified by UV327 also exhibit an LDR of 94.14 dB, rapid response time, and excellent stability exposed to ambient air and 365 nm UV irradiation for a long time. Our work provides a deeper understanding and theoretical guidance for designing efficient Lewis base passivators for UV protection in perovskite optoelectronic devices.

## Supplementary Information

Below is the link to the electronic supplementary material.

Supplementary file1 (DOCX 14863 KB)
