# Supplementary material for: Enhancing Ultraviolet Stability and Operational Durability of Perovskite Photodetectors by Incorporating Chlorine into Thermally-Switchable Tautomeric Passivators
Source: Nanomicro Lett. 2026 Jan 5;18:178. doi: 10.1007/s40820-025-02015-5 (PMC12765762; doi:10.1007/s40820-025-02015-5)
Supplement: Supplementary file 1 — Supplementary file1 (DOCX 14863 KB) [file 40820_2025_2015_MOESM1_ESM.docx]

Supporting Information for

**Enhancing Perovskite Photodetectors' UV Stability and Durability via Chlorine-Incorporated Thermally-Switchable Passivators**

Yong Wang ^1, 2, #^, Guangsheng Liu ^1 ,#^, Feng Lin ^3, #^, Yuqin Hu ^1^, Niu Lai ^1^, Junhong Lv ^1^, Shuming Ye ^1^, Jie Yang ^1^, Rongfei Wang ^1^, Feng Qiu ^1,^ *, Yu Yang ^1, 3^, Wenhua Zhang ^3,^ *, Chong Wang ^1 , 3,^ *

^1^ Yunnan Key Laboratory for Micro/Nano Materials & Technology, School of Materials and Energy, Yunnan University, Kunming 650500, P. R. China

^2^ School of Metallurgy and Materials, Wenshan University, Wenshan 663099, P. R. China

^3^ Yunnan Key Laboratory of Electromagnetic Materials and Devices, School of Materials and Energy, Yunnan University, Kunming 650091, P. R. China

^#^ Yong Wang, Guangsheng Liu, and Feng Lin contributed equally to this work.

* Corresponding authors. E-mail: [cwang@ynu.edu.cn](mailto:cwang@ynu.edu.cn) (Chong Wang); [fengqiu@ynu.edu.cn](mailto:fengqiu@ynu.edu.cn) (Feng Qiu); [20210101@ynu.edu.cn](mailto:20210101@ynu.edu.cn) (Wenhua Zhang )

**S1 Experimental**

**S1.1 Materials**

Lead (II) bromide (PbBr_2_, 99.999%) and Cesium (I) iodide (CsI, 99.999%) were purchased from Yingkou Juntai Solar Photovoltaic Technology Co., Ltd (China). Poly(3-decylthiophene-2,5-diyl), regioregular, electronic grade (P3HT) was acquired from Xi’an Polymer Light Technology Inc (China). The 12% mass fraction SnO_2_ colloidal solution purchased from Xi’an Polymer Light Technology Inc. (China), indium tin oxide (ITO), and fluorine-doped tin oxide (FTO) glass substrates were obtained from Liaoning Shuze Solar Photovoltaic Technology Co., Ltd (China). Dimethyl sulfoxide (DMSO, 99.7%, Extra Dry, with molecular sieves, Water ≤50 ppm) was purchased from Energy Chemical Co., Ltd (China). 2-(2H-Benzo[d][1,2,3]triazol-2-yl)-4,6-di-tert-butylphenol (UV320, 98%) was acquired from Bide Pharmatech CO., Ltd (China). The 2-(3,5-Di-Tert-Butyl-2-Hydroxyphenyl)-5-Chlorobenzotriazole (UV327, 98%) and Dimethyl sulfoxide-d6 (99.8 atom% D) for NMR were obtained from InnoChem company (China).

***S1.2 Device Fabrication***

The FTO glass substrate was cleaned sequentially in an ultrasonic bath using dish soap, deionized water, acetone, isopropyl alcohol, and ethanol, each for 30 minutes. It was then placed in an oven to dry. The cleaned FTO glass was subjected to ozone or plasma cleaning for 30 minutes to enhance its hydrophilicity. Subsequently, a 12% colloidal solution of SnO_2_, diluted 1:3 by volume and filtered through a 0.22 μm filter, was spin-coated onto the FTO substrate at 3000 rpm for 30 seconds. This was followed by annealing at 150 °C for 40 minutes to create a dense SnO_2_ electron transport layer. Subsequently, solutions of UV-320 (0.1, 0.2, 1, and 5 mg/mL) and UV-327 (0.11, 0.22, 1.1, and 5.5 mg/mL) in DMF were spin-coated onto the SnO_2_ substrate at 3000 rpm for 40 seconds. This concentration set was designed to ensure a consistent molar concentration, with the 1 and 1.1 mg/mL UV-320 condition yielding the optimal device performance. PbBr_2_ (367 mg, 1 mmol) and CsI (260 mg, 1 mmol) were dissolved in DMSO and stirred at 60 °C for 2 hours to obtain a clear, bright yellow CsPbIBr_2_ perovskite precursor solution (1 M). This precursor solution was spin-coated onto the SnO_2_ electron transport layer with spin-coating parameters of 1500 rpm for 15 seconds and 3500 rpm for 30 seconds. After standing at 30 °C for 3 minutes, until it turned golden, the film was annealed at 225 °C for 10 minutes to yield a dense CsPbIBr_2_ perovskite film. Subsequently, P3HT (12 mg dissolved in 1 mL of chlorobenzene) was spin-coated onto the perovskite layer at 3000 rpm for 30 seconds and then annealed at 150 °C for 5 minutes. Finally, Ag cathodes (approximately 110 nm) were prepared by thermal evaporation under a 5 × 10^-4^ Pa vacuum, and the active area of the PPDs was 0.18 cm^2^.

**S1.3 Characterizations**

The morphology and grain size of CsPbIBr_2_ perovskite films were measured by Zeiss Gemini 500 field emission scanning electron microscopy (SEM, Germany). The surface morphology of CsPbIBr_2_ perovskite films and the SnO_2_ films was examined by atomic force microscopy model SPI-3800 (AFM, Japan). The crystallinity of CsPbIBr_2_ perovskite films is characterized by X-ray diffraction (XRD, DX-2700BH) with a Cu Kα source. The surface morphology (roughness) of CsPbIBr_2_ perovskite films was examined by atomic force microscopy model SPI-3800 (AFM, Japan). The absorption spectra of perovskite films and the transmittance of ITO glass and the UV320 and UV327 organic matter were characterized by a UV-2600i UV-visible spectrophotometer (UV-vis, SHIMADZU, Japan). The Energy band information of the SnO_2_ film was obtained by ultraviolet photoelectron spectroscopy (AXIS ULTRA DLD, Kratos). The photoluminescence (PL) spectra are obtained through FLS1000, Edinburgh, and the time-resolved photoluminescence (TRPL) measurements were conducted by a near-infrared stable transient absolute quantum yield fluorescence spectroscopy system (Horiba FL-3). The ^1^H and ^13^C nuclear magnetic resonance spectrometer (^1^H NMR, 600 MHz AVANCEIIIHD600, Bruker, Switzerland). The distribution of elements and the chemical interaction between the perovskite and organic functional groups were characterized by X-ray photoelectron spectroscopy (XPS, K-Alpha^+^, Thermo Fisher Scientific, USA) and, Fourier Transform Infrared Spectrometer (FTIR, Nicolet IS10, Thermo Fisher Scientific, USA). Ultraviolet photoelectron spectroscopy (UPS) measurements were performed to determine the valence-band maximum (VBM) of the perovskite modified by UV320 and UV327. These measurements were conducted using a Thermo Fisher Scientific ESCALAB XI^+^ system. In addition, Time-of-flight secondary ion mass spectrometry (TOF-SIMS) depth profiling was performed on an IONTOF M6 system (IONTOF GmbH) to determine the in-depth distribution of UV327 within the perovskite structure. The external quantum efficiency (EQE) of the perovskite thin-film devices was characterized using a solar cell spectral response measurement system (QE-R, ENLITECH). All perovskite thin-film device preparation is done in the glove box (Universal (2440/750/900), Oxygen content＜0.01 ppm, Water content＜0.01 ppm, MIKROUNA, China).

**S1.4 Computational details**

Computational methods: The calculations of ESP were performed by using Gaussian 9 at the B3LYP/6-31G (d) level. The density functional theory (DFT) calculations were conducted by the Vienna ab-Initio Simulation Package (VASP) [S1, S2]. The generalized gradient approximation (GGA) using the Perdew, Burke, and Ernzerhof (PBE) formulation was employed to account for exchange-correlation. The Projected Augmented Wave (PAW) potentials were used to describe the ionic cores and to explain valence electrons using a plane wave basis set with a kinetic energy cutoff of 450 eV. During structure optimization, the conjugate-gradient method was performed, and convergence criteria for energy and force were set to 5×10^-5^ eV and 3×10^-2^ eV Å^-1^, respectively. In addition, the computational setup for all adsorption energy calculations incorporated a 3×3×2 supercell combined with a 2×2×1 k-point grid. The Grimm’s DFT-D3 correction was considered to describe the interactions between slab models and organics [S3].

**S2 Supplementary Figures and Tables**

**Fig. S1** The Bader charge transfer on the oxygen atoms of both -OH and -C=O groups after the adsorption of UV320, UV327, K-UV320, and K-UV327 molecules onto the perovskite

**Figure S2** The ^1^H NMR spectra of the UV320 molecule before and after being added to the perovskite upon heating, respectively

**Fig. S3** The ^1^H NMR spectra of the UV327 molecule before and after being added to the perovskite upon heating, respectively

**Fig. S4** The ^1^H NMR spectra of the UV320 molecule before and after being added to the perovskite upon ultraviolet radiation, respectively

**Fig. S5** The ^1^H NMR spectra of the UV327 molecule before and after being added to the perovskite upon ultraviolet radiation, respectively

**Fig. S6** Analysis of the tautomeric distribution (enol vs. keto forms) in UV320-perovskite mixtures via ^1^H NMR spectroscopy under heating and UV irradiation

**Fig. S7** Analysis of the tautomeric distribution (enol vs. keto forms) in UV327-perovskite mixtures via ^1^H NMR spectroscopy under heating and UV irradiation

The mixed solutions of UV320 and UV327 with perovskite in dimethyl sulfoxide-d_6_ (DMSO-d_6_) were prepared, and their ^1^H NMR spectra were acquired under three conditions: the initial state, after heating, and after UV irradiation (**Fig.**s S7 and S8). The approximate content of the keto form under these conditions was estimated by integration of the characteristic peak areas. For UV320, the contents were determined to be 7%, 14%, and 3%, respectively, while for UV327, they were 9%, 17%, and 4%. The results indicate that heating promotes the conversion of the enol form to the keto form, and the presence of a Cl atom appears to facilitate this process. Furthermore, after UV irradiation, the spectral profiles revert to their original shapes, accompanied by a decrease in the keto form content, demonstrating the reversible process described in the main text. In addition to external stimuli such as heating and UV irradiation, the role of the solvent in influencing this equilibrium must also be considered.

Furthermore, the potential influence of the DMSO-d_6_ solvent on the tautomeric equilibrium in our system is an important point. DMSO, a strongly polar aprotic solvent, is an excellent hydrogen-bond acceptor. It tends to solvate the hydroxyl group of the enol form, which could competitively disrupt the intramolecular hydrogen bond and potentially shift the equilibrium toward the keto form. Therefore, the tautomeric behavior observed in this work should be understood as specific to the DMSO environment.

**Fig. S8** Charge distribution of molecules (**a**) UV320. (**b**) UV327. (**c**) K-UV320. (**d**) K-UV327

**Fig. S9** The mass spectrum of the TOF-SIMS test

**Fig. S10** (**a**) TOF-SIMS spectra of Cl^-^. (**b**) The 3D render of Cl^-^ in TOF-SIMS spectra

**Fig. S11** The AFM images of (**a**) control, (**b**) UV320-treated, and (**c**) UV320-treated perovskite films. (**d-f**) The AFM image of PVK, PVK-UV320, and PVK-UV327 films spans the corresponding height contour along the entire surface, respectively

**Fig. S12** The optical band gap of the three types of perovskite film

**Fig. S13** The transmittance of ITO with and without UV320 and UV327 treatment

**Fig. S14** The AFM image of (**a**) SnO_2_, (**b**) SnO_2_-UV320, and (**c**) SnO_2_-UV327 films spans the corresponding height contour along the entire surface

**Fig. S15** The surface contact potential difference (*V_CPD_*) of (a) SnO_2_, (b) SnO_2_-UV320, and (c) SnO_2_-UV327 films

**Fig. S16** UPS spectra of (**a**) PVK, (**b**) PVK-UV320, and (**c**) PVK-UV327 films

**Fig. S17** The XPS spectra for the perovskite films without and with UV320 and UV327 treatment (**a**) Full spectrum, (**b**) Cs 3d, (**c**) Br 3d

**Fig. S18** The O 1s spectra for the SnO_2_ films without and with UV320 and UV327 treatment

**Fig. S19** The XPS spectra for the SnO_2_ films without and with UV320 and UV327 treatment (**a**) N 1s in UV320. (**b**) N 1s in UV327. (**c**) C 1s in UV320. (**d**) C 1s in UV327. (**e**) Cl 2p in UV327

**Fig. S20** FTIR spectra: (**a**) -C=N and (**b**) -C=O of UV320 and UV-320PbI_2_. (**c**) -C=O and (**d**) -C=N of UV320 and UV320-Sn^4+^. (**e**) -C=N of UV327 and UV327-Sn^4+^

**Fig. S21** The photocurrent response curve of PD320 at a frequency of 3907 Hz

**Table S1** The fitting parameters of the TRPL spectra for bare control device and UV320 and UV327-modified films without SnO_2_

| Sample | *τ*ave (ns) | *τ*1 (ns) | *А*1 (%) | *τ*2 (ns) | *А*2 (%) |
| --- | --- | --- | --- | --- | --- |
| FTO/PVK | 370.76 | 201.13 | 0.12 | 858.30 | 0.0097 |
| FTO/UV320-PVK | 460.64 | 242.74 | 0.142 | 1630.39 | 0.0039 |
| FTO/UV327-PVK | 470.71 | 149.687 | 0.247 | 1179.41 | 0.0142 |

**Table S2** The fitting parameters of the TRPL spectra for control and UV-320 and UV-327 modified films

| Sample | *τ*ave (ns) | *τ*1 (ns) | *А*1 (%) | *τ*2 (ns) | *А*2 (%) |
| --- | --- | --- | --- | --- | --- |
| FTO/SnO_2_/PVK | 863.94 | 168.54 | 764.13 | 1055.54 | 442.81 |
| FTO/UVSnO_2_/320-PVK | 825.25 | 148.96 | 445.98 | 922.24 | 500.24 |
| FTO/SnO_2_/UV327-PVK | 795.29 | 143.41 | 734.46 | 961.73 | 428.95 |

**Table S3** Summary of the Performance Parameters for the Self-Powered CsPbI_x_Br_3–x_ PD Devices

| Type of device | Dark current (nA) | Responsivity  (A/W) | Detectivity (10^12^ jones) | Refs. |
| --- | --- | --- | --- | --- |
| FTO/SnO_2_-Gua/CsPbIBr_2_/P3HT/Ag | **0.057** | **0.30** | **29.6** | **This work** |
| FTO/TiO_2_/S-CsPbIBr_2_/Carbon | 50 | 0.33 | 3.9 | [S4] |
| FTO/PEI-CsPbIBr_2_/carbon | 2.02 | 0.32 | 3.7 | [S5] |
| FTO/TiO_2_/PEI-CsPbIBr_2_/carbon | 0.312 | 0.20 | 6.0 | [S6] |
| ITO/SnO_2_/PEGCsPbI_2_Br/PTAA/MoO_3_/Ag | 1 | 0.43 | 0.2 | [S7] |
| FTO/SnO_2_/CsPbI_3_-Yb^3+^/Spiro-OMETAD-Au | / | 0.12 | 4.6 | [S8] |
| FTO/SnO_2_/MAPbI_3_-CsPbI_3_QDs/Carbon | 0.17 | 0.37 | 4.7 | [S9] |
| FTO/c-TiO_2_/CsPbBr_3_-CSA/Carbon | 5 | 0.0015 | 0.002 | [S10] |
| FTO/TiO_2_/CsPbCl_3_-BMMImCl/Carbon | 10.5 | 0.21 | 6.2 | [S11] |
| FTO/TiO_2_/CsPbIBr_2_ (AgI)/Spiro-OMETAD/Au | 9700 | 0.43 | 0.3 | [S12] |
| FTO/TiO_2_/CsPbIBr_2_/Spiro-OMETAD/Au | 44400 | 0.38 | 0.1 | [S12] |
| FTO/NiO_x_/CsPbBr_3_/TiO_X_/Ag | / | 0.11 | 2.2 | [S13] |
| FTO/SnO_2_/CsPb_2_Br_5_-CsPbBr_3_/ Carbon | 1 | 0.11 | 1.4 | [S14] |

**Supplementary References**

1. G. Kresse, J. Furthmüller, Efficiency of ab-initio total energy calculations for metals and semiconductors using a plane-wave basis set. Comput. Mater. Sci. **6**(1), 15–50 (1996). <https://doi.org/10.1016/0927-0256(96)00008-0>
2. G. Kresse, J. Furthmüller, Efficient iterative schemes for *ab initio* total-energy calculations using a plane-wave basis set. Phys. Rev. B **54**(16), 11169–11186 (1996). <https://doi.org/10.1103/physrevb.54.11169>
3. S. Grimme, J. Antony, S. Ehrlich, H. Krieg, A consistent and accurate *ab initio* parametrization of density functional dispersion correction (DFT-D) for the 94 elements H-Pu. J. Chem. Phys. **132**(15), 154104 (2010). <https://doi.org/10.1063/1.3382344>
4. J. Du, J. Duan, X. Yang, Q. Zhou, Y. Duan et al., Reducing defect of inorganic perovskite film by sulphur-containing Lewis base for robust photodetectors. J. Energy Chem. **61**, 163–169 (2021). <https://doi.org/10.1016/j.jechem.2021.02.004>
5. Z. Zhang, W. Zhang, Q. Jiang, Z. Wei, M. Deng et al., Toward high-performance electron/hole-transporting-layer-free, self-powered CsPbIBr_2_ photodetectors *via* interfacial engineering. ACS Appl. Mater. Interfaces **12**(5), 6607–6614 (2020). <https://doi.org/10.1021/acsami.9b19075>
6. Z. Zhang, W. Zhang, Z. Wei, Q. Jiang, M. Deng et al., Dipole-templated homogeneous grain growth of CsPbIBr_2_ films for efficient self-powered, all-inorganic photodetectors. Sol. Energy **209**, 371–378 (2020). <https://doi.org/10.1016/j.solener.2020.09.021>
7. J. Li, G. Zhang, Z. Zhang, J. Li, Z. Uddin et al., Defect passivation *via* additive engineering to improve photodetection performance in CsPbI_2_Br perovskite photodetectors. ACS Appl. Mater. Interfaces **13**(47), 56358–56365 (2021). <https://doi.org/10.1021/acsami.1c19323>
8. Z. Fang, N. Ding, W. Xu, T. Wang, Y. Wang et al., Lanthanide ion doping enabling highly sensitive and stable all-inorganic CsPbI_2_Br perovskite photodetectors. J. Mater. Chem. C **11**(12), 4049–4055 (2023). <https://doi.org/10.1039/D2TC05543K>
9. H. Zhou, M. Chen, C. Liu, R. Zhang, J. Li et al., Interfacial passivation of CsPbI(3) quantum dots improves the performance of hole-transport-layer-free perovskite photodetectors. Discov. Nano **18**(1), 11 (2023). <https://doi.org/10.1186/s11671-023-03793-w>
10. M. Dhakshnamoorthy, A. Kathirvel, S.M. Raj, V.R. Ancha, M. Abebe et al., Self-powered white light photodetector with enhanced photoresponse using camphor sulphonic acid treated CsPbBr_3_ perovskite in carbon matrix. Mater. Lett. **341**, 134250 (2023). <https://doi.org/10.1016/j.matlet.2023.134250>
11. S. Cheng, X. Zheng, Z. Hou, R. Hu, S. Jiang et al., Passivating the vacancy defects of CsPbCl_3_ polycrystalline films by a Cl-containing ionic liquid for self-powered, charge-transport-layer-free UV photodetectors. J. Mater. Chem. C **10**(14), 5693–5706 (2022). <https://doi.org/10.1039/D2TC00080F>
12. V.O. Eze, G.R. Adams, L. Braga Carani, R.J. Simpson, O.I. Okoli, Enhanced inorganic CsPbIBr_2_ perovskite film for a sensitive and rapid response self-powered photodetector. J. Phys. Chem. C **124**(38), 20643–20653 (2020). <https://doi.org/10.1021/acs.jpcc.0c04144>
13. F. Guo, J. Wang, Y. Li, S. Yu, X. Xu et al., Postripening fabrication and self-driven narrowband photoresponse of large-grain, phase-pure CsPbBr_3_ films. Sol. RRL **6**(12), 2200828 (2022). <https://doi.org/10.1002/solr.202200828>
14. R. Liu, J. Zhang, H. Zhou, Z. Song, Z. Song et al., Solution-processed high-quality cesium lead bromine perovskite photodetectors with high detectivity for application in visible light communication. Adv. Opt. Mater. **8**(8), 1901735 (2020). <https://doi.org/10.1002/adom.201901735>
